# *N*-Acetyl-3,5-dibromo-l-tyrosine hemihydrate

**DOI:** 10.1107/S1600536812032928

**Published:** 2012-08-01

**Authors:** Pakorn Bovonsombat, John Snyder, Francesco Caruso, Miriam Rossi

**Affiliations:** a Mahidol University International College, Mahidol University, Salaya Campus, Nakorn Pathom 73170, Thailand; b Department of Chemistry, Vassar College, Poughkeepsie, NY 12604-0484, USA; c Istituto Chimica Biomolecolare CNR, P.le Aldo Moro 5, 00185, Rome, Italy

## Abstract

The title compound, C_11_H_11_Br_2_NO_4_·0.5H_2_O, was prepared by an electrophilic bromination of *N*-acetyl-l-tyrosine in acetonitrile at room temperature. The two independent mol­ecules do not differ substanti­ally and a mol­ecule of water completes the asymmetric unit. The synthesis of the title compound does not modify the stereochemical center, as shown by the absolute configuration found in this crystal structure. Comparison with the non-bromo starting material differs mainly by rotation features. For instance the H(methine)—C(chiral center)—C(methyl­ene)—C(*ipso*) is 173.0 (2)° torsion angle in one mol­ecule and 177.3 (2)° in the other, indicating a *trans* arrangement. This is in contrast with approximately 50° in the starting material. A short inter­molecular Br⋯Br separation is observed [3.2938 (3) Å]. The molecules in the crystal are connected *via* a network of hydrogen bonds through an N—H⋯O hydrogen bond between the hydroxy group of the phenol of the tyrosine and the N—H of the amide of the other molecule and an O—H⋯O hydrogen bond between the hydroxy group of the carboxylic acid and the oxygen of the carbonyl of the amide.

## Related literature

*N*-Acetyl-3,5-dibromo-l-tyrosine is a substrate of biological inter­est, for instance it is involved in the synthesis of isodityrosine unit, which has been found in numerous biologically active natural products that include K-13, OF4949 and vancomycin family of glycopeptide anti­biotics. For the synthesis and specific optical activity of the title compound, see: Bovonsombat *et al.* (2008 ▶); Dewitt & Ingersoll (1951 ▶). For the synthesis of isodityrosine, see: Guo *et al.* (1997 ▶). For the structure of Bastadin 6, see: Kazlauskas *et al.* (1980 ▶, 1981 ▶). For the structure of the starting material, *N*-acetyl-l-tyrosine, see: Koszelak & van der Helm (1981 ▶). For structures with similarly short Br⋯Br contacts, see Quast *et al.* (1995 ▶).
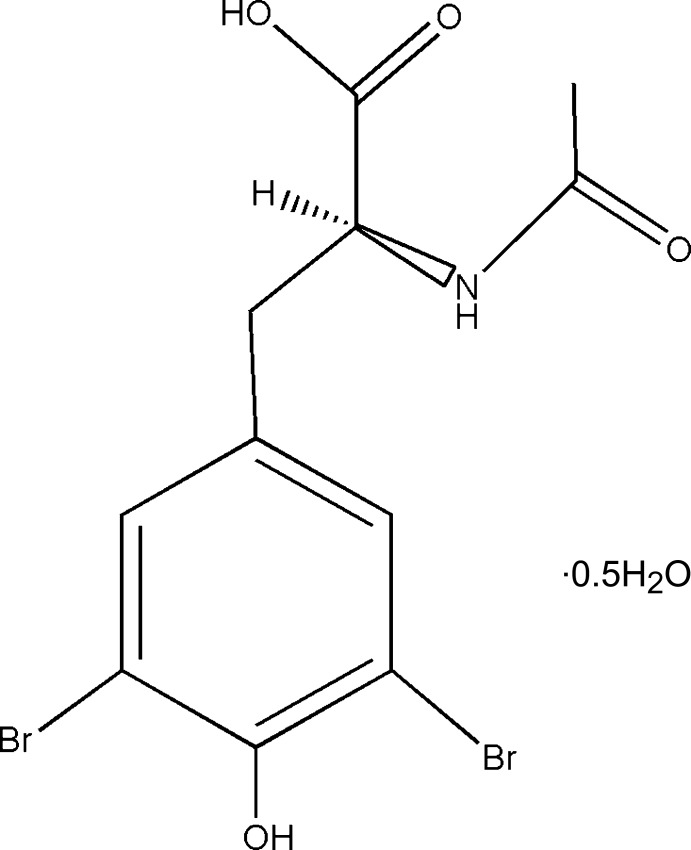


## Experimental

### 

#### Crystal data

C_11_H_11_Br_2_NO_4_·0.5H_2_O
*M* *_r_* = 390.02
Monoclinic, 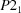

*a* = 7.1095 (3) Å
*b* = 22.5186 (9) Å
*c* = 8.6486 (4) Å
β = 105.946 (1)°
*V* = 1331.3 (1) Å^3^
*Z* = 4
Mo *K*α radiation
μ = 6.10 mm^−1^
*T* = 125 K
0.23 × 0.17 × 0.05 mm

#### Data collection

Bruker SMART CCD area-detector diffractometer
Absorption correction: multi-scan (*SADABS*; Bruker 2007 ▶) *T*_min_ = 0.334, *T*_max_ = 0.750
18331 measured reflections
7067 independent reflections
6449 reflections with *I* > 2σ(*I*)
*R*_int_ = 0.030

#### Refinement

*R*[*F* ^2^ > 2σ(*F* ^2^)] = 0.022
*wR*(*F* ^2^) = 0.048
*S* = 0.92
7067 reflections
361 parameters
17 restraints
H atoms treated by a mixture of independent and constrained refinement
Δρ_max_ = 0.41 e Å^−3^
Δρ_min_ = −0.34 e Å^−3^
Absolute structure: Flack (1983 ▶), 3399 Friedel pairs
Flack parameter: 0.005 (5)

### 

Data collection: *SMART* (Bruker, 2007 ▶); cell refinement: *SAINT* (Bruker, 2007 ▶); data reduction: *SAINT*; program(s) used to solve structure: *SHELXS97* (Sheldrick, 2008 ▶); program(s) used to refine structure: *SHELXL97* (Sheldrick, 2008 ▶); molecular graphics: *SHELXTL* (Sheldrick, 2008 ▶); software used to prepare material for publication: *SHELXTL*.

## Supplementary Material

Crystal structure: contains datablock(s) global, I. DOI: 10.1107/S1600536812032928/bg2470sup1.cif

Structure factors: contains datablock(s) I. DOI: 10.1107/S1600536812032928/bg2470Isup2.hkl

Supplementary material file. DOI: 10.1107/S1600536812032928/bg2470Isup3.cml

Additional supplementary materials:  crystallographic information; 3D view; checkCIF report

## Figures and Tables

**Table 1:** Hydrogen-bond geometry (Å, °)

*D*—H⋯*A*	*D*—H	H⋯*A*	*D*⋯*A*	*D*—H⋯*A*
N2—H2*N*⋯O1^i^	0.85 (1)	2.11 (1)	2.945 (3)	169 (3)
O7—H7*O*⋯O1*W* ^ii^	0.83 (1)	1.78 (1)	2.607 (3)	171 (3)
O1*W*—H1*W*⋯O4^iii^	0.84 (1)	2.11 (2)	2.814 (3)	142 (3)
O1*W*—H2*W*⋯O2^iv^	0.83 (1)	2.01 (1)	2.836 (3)	172 (4)
N1—H1*N*⋯O5^v^	0.85 (1)	2.31 (1)	3.152 (3)	170 (2)
O3—H3*O*⋯O8^iv^	0.83 (1)	1.76 (1)	2.573 (2)	165 (3)
O5—H5*O*⋯Br4	0.83 (1)	2.73 (3)	3.1096 (16)	110 (2)
O5—H5*O*⋯O4^vi^	0.83 (1)	1.96 (1)	2.732 (2)	156 (3)
O1—H1*O*⋯O6^vii^	0.83 (1)	2.06 (2)	2.750 (2)	141 (3)
O1—H1*O*⋯Br3	0.83 (1)	2.61 (3)	3.0777 (17)	117 (2)

Symmetry codes: (i) 
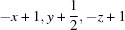
; (ii) 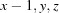; (iii) 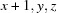; (iv) 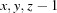; (v) 
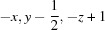
; (vi) 
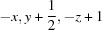
; (vii) 
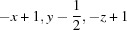
.

**Fig. 1.**
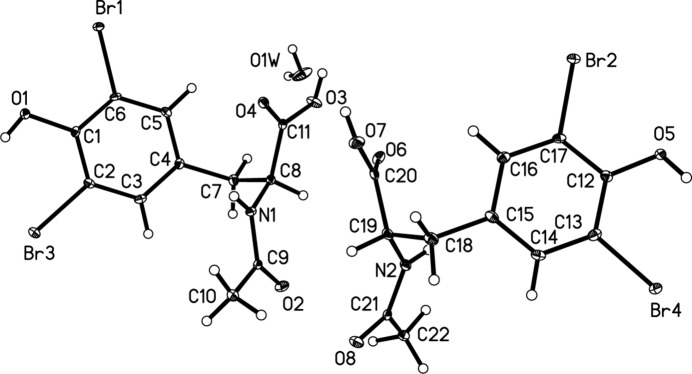
Ellipsoid plot.

## supplementary crystallographic information

### Comment

We report here the structure of the title compound (I). The 3,5-dibromo-*L*-tyrosine moiety is an integral part of Bastadin 6, a secondary metabolite possessing antibacterial activity against Gram-positve bacteria, extracted from Verongrid sponge *Ianthella basta*. Geometrical parameters of both independent molecules do not differ substantially. Comparison with the non-bromo starting material differs mainly by rotation features. For instance the torsion angle H(methine)-C(chiral center)-*C*(methylene)-C(*ipso*) is 173.0 (2)° in one molecule, 177.3 (2)° in the other, indicating a *trans* arrangement. This is in contrast with 49.5 Å in the starting material (Koszelak & van der Helm, 1981). The intermolecular Br1—Br2 separation of 3.2938 (3) Å is markedly shorter than the corresponding van der Waals Br—Br separation. A similar Br–Br short separation (Br—Br = 3.295 (2) Å)) is found in *exo*-4,*exo*-8-dibromo-3,7-bis(phenylsulfonyl)bicyclo(3.3.1)nona-2,6-diene (Quast *et al.* (1995)). A related exploration in the CSD displays 2948 hits shorter than 3.70?Å. In about 200 hits, related Br—Br separations shorter than 3.30?Å are found, and many of them include isolated Br atoms.

### Experimental

To a stirring solution of *N*-acetyl-*L*-tyrosine (1 mmol) in 20 ml of solvent, 2 mmol (2.0 equivalents) of *N*-bromosuccinimide were added in one portion. The reaction was left to stir at room temperature for 18 h. For the work up, the organic solution was diluted with 80 ml of ethyl acetate and washed three times (20 ml aliquots) with a 5% aqueous solution of Na_2_S_2_O_3_, followed by three washes with water (20 ml aliquots) and lastly with brine. After evaporation of the solvents under vacuum, the solid was subjected to silica gel chromatography (EtOAc/hexanes/CH_3_OH (6:3:1) and 0.1% acetic acid). The product was recrystallized from water. NMR data are consistent with those reported in the literature, Bovonsombat *et al.* (2008). For the crystal structure experiment, the crystals were obtained from a partially dried solution of the title compound dissolved in methanol (10 mg in 2 ml of methanol).

### Refinement

All the H atoms were clearly seen in a difference Fourier but they were treated differently in refinement: C—H's were re-positioned at their expected locations, and allowed to ride both in coordinates (C—H = 0.93/0.98 Å), as well as in isotropic displacement factors [*U*_iso_(H) = 1.2-1.5× *U*_eq_(host)]; those attached to N and O were refined with restrained distance ( N—H = O—H: 0.85 (1)Å) and free *U*_iso_(H).

### Crystal data

**Table:**

C_11_H_11_Br_2_NO_4_·0.5H_2_O	*F*(000) = 764
*M**_r_* = 390.02	*D*_x_ = 1.946 Mg m^−^^3^
Monoclinic, *P*2_1_	Mo *K*α radiation, λ = 0.71073 Å
Hall symbol: P 2yb	Cell parameters from 9469 reflections
*a* = 7.1095 (3) Å	θ = 2.5–30.2°
*b* = 22.5186 (9) Å	µ = 6.10 mm^−^^1^
*c* = 8.6486 (4) Å	*T* = 125 K
β = 105.946 (1)°	Plate, colourless
*V* = 1331.3 (1) Å^3^	0.23 × 0.17 × 0.05 mm
*Z* = 4	

### Data collection

**Table:**

Bruker SMART CCD area-detector diffractometer	7067 independent reflections
Radiation source: fine-focus sealed tube	6449 reflections with *I* > 2σ(*I*)
Graphite monochromator	*R*_int_ = 0.030
phi and ω scans	θ_max_ = 29.1°, θ_min_ = 1.8°
Absorption correction: multi-scan (*SADABS*; Bruker 2007)	*h* = −9→9
*T*_min_ = 0.334, *T*_max_ = 0.750	*k* = −30→30
18331 measured reflections	*l* = −11→11

### Refinement

**Table:**

Refinement on *F*^2^	Secondary atom site location: difference Fourier map
Least-squares matrix: full	Hydrogen site location: inferred from neighbouring sites
*R*[*F*^2^ > 2σ(*F*^2^)] = 0.022	H atoms treated by a mixture of independent and constrained refinement
*wR*(*F*^2^) = 0.048	*w* = 1/[σ^2^(*F*_o_^2^)]
*S* = 0.92	(Δ/σ)_max_ = 0.003
7067 reflections	Δρ_max_ = 0.41 e Å^−^^3^
361 parameters	Δρ_min_ = −0.34 e Å^−^^3^
17 restraints	Absolute structure: Flack (1983), 3399 Friedel pairs
Primary atom site location: structure-invariant direct methods	Flack parameter: 0.005 (5)

### Special details

**Table:**

Geometry. All esds (except the esd in the dihedral angle between two l.s. planes) are estimated using the full covariance matrix. The cell esds are taken into account individually in the estimation of esds in distances, angles and torsion angles; correlations between esds in cell parameters are only used when they are defined by crystal symmetry. An approximate (isotropic) treatment of cell esds is used for estimating esds involving l.s. planes.

### Fractional atomic coordinates and isotropic or equivalent isotropic displacement parameters (Å^2^)

**Table:**

	*x*	*y*	*z*	*U*_iso_*/*U*_eq_	
Br1	0.52379 (3)	0.027010 (11)	0.09624 (3)	0.02171 (6)	
Br2	−0.20768 (3)	0.529982 (10)	0.26246 (3)	0.02077 (6)	
Br3	0.70806 (4)	−0.044388 (11)	0.74402 (3)	0.02526 (7)	
Br4	−0.01205 (4)	0.612664 (12)	0.90382 (3)	0.02911 (7)	
C1	0.6076 (3)	0.00179 (10)	0.4288 (3)	0.0144 (4)	
C2	0.6679 (3)	0.01750 (11)	0.5896 (3)	0.0170 (5)	
C3	0.7095 (3)	0.07588 (11)	0.6382 (3)	0.0181 (5)	
H3	0.7475	0.0850	0.7473	0.022*	
C4	0.6948 (3)	0.12065 (11)	0.5257 (3)	0.0160 (4)	
C5	0.6346 (3)	0.10533 (11)	0.3629 (3)	0.0176 (5)	
H5	0.6229	0.1346	0.2850	0.021*	
C7	0.7528 (3)	0.18382 (10)	0.5762 (3)	0.0186 (5)	
H7B	0.8205	0.2003	0.5028	0.022*	
H7A	0.8446	0.1831	0.6824	0.022*	
C8	0.5815 (3)	0.22554 (10)	0.5802 (3)	0.0168 (5)	
H8	0.6378	0.2634	0.6268	0.020*	
C9	0.5205 (4)	0.20950 (11)	0.8411 (3)	0.0217 (5)	
C10	0.3882 (4)	0.18128 (12)	0.9301 (3)	0.0320 (7)	
H10C	0.2806	0.1622	0.8546	0.048*	
H10A	0.3392	0.2113	0.9876	0.048*	
H10B	0.4606	0.1524	1.0047	0.048*	
C12	−0.1141 (3)	0.56247 (11)	0.5916 (3)	0.0168 (5)	
C13	−0.0614 (3)	0.54956 (12)	0.7546 (3)	0.0202 (5)	
C17	−0.1403 (3)	0.51408 (11)	0.4856 (3)	0.0163 (5)	
C16	−0.1215 (3)	0.45657 (12)	0.5400 (3)	0.0184 (5)	
H16	−0.1406	0.4256	0.4661	0.022*	
C14	−0.0419 (3)	0.49135 (12)	0.8107 (3)	0.0202 (5)	
H14	−0.0068	0.4843	0.9209	0.024*	
C11	0.4499 (3)	0.23828 (10)	0.4122 (3)	0.0156 (5)	
C15	−0.0741 (3)	0.44352 (11)	0.7047 (3)	0.0179 (5)	
C20	0.1074 (4)	0.33012 (10)	0.5784 (3)	0.0199 (5)	
C19	0.1059 (3)	0.34237 (10)	0.7516 (3)	0.0195 (5)	
H19	0.0927	0.3038	0.8002	0.023*	
C21	0.3886 (3)	0.34854 (10)	0.9878 (3)	0.0169 (5)	
C22	0.5904 (4)	0.37301 (11)	1.0570 (3)	0.0205 (5)	
H22C	0.6055	0.4087	1.0009	0.031*	
H22B	0.6853	0.3443	1.0454	0.031*	
H22A	0.6097	0.3817	1.1689	0.031*	
C18	−0.0683 (4)	0.38071 (11)	0.7657 (3)	0.0224 (5)	
H18B	−0.1881	0.3610	0.7075	0.027*	
H18A	−0.0672	0.3821	0.8781	0.027*	
C6	0.5923 (3)	0.04698 (11)	0.3168 (3)	0.0161 (5)	
N1	0.4654 (3)	0.20206 (9)	0.6814 (2)	0.0177 (4)	
H1N	0.371 (3)	0.1796 (10)	0.634 (3)	0.021*	
N2	0.2930 (3)	0.36671 (9)	0.8409 (2)	0.0189 (4)	
H2N	0.334 (4)	0.3930 (9)	0.788 (3)	0.023*	
O1	0.5651 (2)	−0.05488 (7)	0.3754 (2)	0.0185 (4)	
H1O	0.611 (4)	−0.0801 (9)	0.445 (2)	0.022*	
O2	0.6678 (3)	0.23827 (9)	0.9097 (2)	0.0316 (4)	
O3	0.5303 (3)	0.27618 (9)	0.3358 (2)	0.0269 (4)	
H3O	0.446 (3)	0.2834 (13)	0.2502 (19)	0.032*	
O4	0.2913 (2)	0.21517 (7)	0.35608 (19)	0.0187 (3)	
O5	−0.1397 (2)	0.61836 (8)	0.53021 (19)	0.0206 (4)	
H5O	−0.166 (4)	0.6438 (9)	0.590 (3)	0.025*	
O6	0.2455 (3)	0.33961 (8)	0.5251 (2)	0.0257 (4)	
O7	−0.0617 (3)	0.30763 (9)	0.4961 (2)	0.0284 (4)	
H7O	−0.051 (4)	0.2962 (13)	0.4076 (19)	0.034*	
O8	0.3140 (2)	0.31234 (8)	1.0623 (2)	0.0234 (4)	
O1W	0.9302 (3)	0.26971 (13)	0.2095 (3)	0.0520 (7)	
H1W	1.038 (3)	0.2551 (16)	0.207 (4)	0.062*	
H2W	0.847 (4)	0.2589 (17)	0.127 (3)	0.062*	

### Atomic displacement parameters (Å^2^)

**Table:**

	*U*^11^	*U*^22^	*U*^33^	*U*^12^	*U*^13^	*U*^23^
Br1	0.02863 (13)	0.02049 (12)	0.01247 (12)	0.00333 (11)	−0.00033 (10)	−0.00168 (10)
Br2	0.02392 (13)	0.02328 (14)	0.01258 (12)	0.00015 (11)	0.00074 (10)	0.00066 (10)
Br3	0.03634 (16)	0.02193 (14)	0.01756 (13)	−0.00037 (11)	0.00752 (11)	0.00566 (10)
Br4	0.04323 (17)	0.02575 (14)	0.01522 (13)	0.01038 (12)	0.00278 (11)	−0.00298 (10)
C1	0.0122 (10)	0.0121 (11)	0.0188 (11)	0.0018 (9)	0.0038 (9)	−0.0018 (9)
C2	0.0181 (11)	0.0186 (13)	0.0149 (10)	0.0020 (9)	0.0054 (9)	0.0034 (9)
C3	0.0175 (11)	0.0231 (13)	0.0131 (11)	0.0023 (10)	0.0029 (9)	−0.0034 (9)
C4	0.0124 (10)	0.0150 (11)	0.0197 (11)	0.0008 (9)	0.0027 (9)	−0.0009 (9)
C5	0.0165 (11)	0.0185 (12)	0.0167 (11)	0.0022 (9)	0.0027 (9)	0.0045 (9)
C7	0.0151 (11)	0.0160 (11)	0.0217 (12)	−0.0017 (9)	−0.0002 (9)	0.0001 (9)
C8	0.0202 (11)	0.0128 (11)	0.0148 (11)	−0.0025 (9)	0.0006 (9)	−0.0012 (8)
C9	0.0361 (15)	0.0121 (11)	0.0131 (11)	0.0057 (10)	0.0004 (10)	−0.0008 (9)
C10	0.059 (2)	0.0202 (14)	0.0182 (13)	−0.0014 (13)	0.0134 (13)	0.0011 (11)
C12	0.0135 (11)	0.0204 (13)	0.0163 (12)	0.0031 (9)	0.0039 (9)	0.0021 (10)
C13	0.0166 (12)	0.0259 (13)	0.0174 (12)	0.0025 (10)	0.0033 (10)	−0.0054 (10)
C17	0.0133 (10)	0.0229 (14)	0.0111 (10)	0.0001 (9)	0.0006 (8)	−0.0010 (9)
C16	0.0143 (11)	0.0225 (12)	0.0167 (11)	−0.0016 (10)	0.0015 (9)	−0.0029 (10)
C14	0.0178 (11)	0.0289 (14)	0.0127 (11)	0.0020 (10)	0.0020 (9)	0.0031 (10)
C11	0.0188 (12)	0.0109 (10)	0.0171 (11)	0.0014 (9)	0.0046 (9)	−0.0015 (9)
C15	0.0123 (10)	0.0222 (13)	0.0181 (11)	0.0014 (9)	0.0024 (9)	0.0055 (9)
C20	0.0236 (13)	0.0103 (11)	0.0202 (12)	0.0010 (9)	−0.0031 (10)	0.0023 (9)
C19	0.0230 (12)	0.0146 (11)	0.0176 (12)	−0.0054 (9)	0.0000 (10)	0.0043 (9)
C21	0.0220 (12)	0.0115 (11)	0.0163 (11)	0.0044 (9)	0.0039 (9)	−0.0015 (9)
C22	0.0220 (12)	0.0194 (13)	0.0174 (11)	0.0022 (10)	0.0007 (9)	0.0013 (10)
C18	0.0221 (12)	0.0233 (13)	0.0210 (12)	−0.0035 (10)	0.0046 (10)	0.0047 (10)
C6	0.0158 (11)	0.0209 (12)	0.0099 (11)	0.0023 (9)	0.0010 (9)	−0.0034 (9)
N1	0.0236 (11)	0.0166 (10)	0.0116 (9)	−0.0030 (8)	0.0024 (8)	−0.0016 (8)
N2	0.0218 (10)	0.0149 (10)	0.0169 (10)	−0.0059 (8)	0.0002 (8)	0.0027 (8)
O1	0.0217 (9)	0.0142 (9)	0.0180 (9)	0.0010 (7)	0.0026 (7)	0.0005 (7)
O2	0.0394 (12)	0.0297 (11)	0.0183 (9)	−0.0018 (9)	−0.0047 (8)	−0.0065 (8)
O3	0.0218 (9)	0.0353 (11)	0.0198 (9)	−0.0082 (8)	−0.0008 (7)	0.0117 (8)
O4	0.0178 (8)	0.0163 (8)	0.0192 (8)	−0.0024 (7)	0.0007 (7)	0.0020 (7)
O5	0.0273 (9)	0.0184 (9)	0.0156 (9)	0.0072 (8)	0.0050 (7)	0.0001 (7)
O6	0.0281 (10)	0.0221 (10)	0.0257 (10)	−0.0043 (8)	0.0053 (8)	−0.0087 (8)
O7	0.0292 (10)	0.0302 (11)	0.0203 (9)	−0.0120 (8)	−0.0024 (8)	−0.0030 (8)
O8	0.0240 (9)	0.0275 (10)	0.0171 (8)	−0.0023 (7)	0.0032 (7)	0.0057 (7)
O1W	0.0294 (12)	0.0756 (18)	0.0375 (13)	0.0269 (12)	−0.0135 (10)	−0.0321 (12)

### Geometric parameters (Å, º)

**Table:**

Br1—C6	1.889 (2)	C17—C16	1.372 (4)
Br2—C17	1.890 (2)	C16—C15	1.402 (3)
Br3—C2	1.897 (2)	C16—H16	0.9300
Br4—C13	1.887 (3)	C14—C15	1.392 (4)
C1—O1	1.362 (3)	C14—H14	0.9300
C1—C2	1.384 (3)	C11—O4	1.215 (3)
C1—C6	1.388 (3)	C11—O3	1.304 (3)
C2—C3	1.387 (3)	C15—C18	1.506 (3)
C3—C4	1.385 (3)	C20—O6	1.213 (3)
C3—H3	0.9300	C20—O7	1.318 (3)
C4—C5	1.398 (3)	C20—C19	1.526 (3)
C4—C7	1.512 (3)	C19—N2	1.450 (3)
C5—C6	1.382 (3)	C19—C18	1.542 (3)
C5—H5	0.9300	C19—H19	0.9800
C7—C8	1.546 (3)	C21—O8	1.245 (3)
C7—H7B	0.9700	C21—N2	1.330 (3)
C7—H7A	0.9700	C21—C22	1.499 (3)
C8—N1	1.457 (3)	C22—H22C	0.9600
C8—C11	1.524 (3)	C22—H22B	0.9600
C8—H8	0.9800	C22—H22A	0.9600
C9—O2	1.236 (3)	C18—H18B	0.9700
C9—N1	1.339 (3)	C18—H18A	0.9700
C9—C10	1.510 (4)	N1—H1N	0.852 (7)
C10—H10C	0.9600	N2—H2N	0.847 (7)
C10—H10A	0.9600	O1—H1O	0.829 (7)
C10—H10B	0.9600	O3—H3O	0.832 (7)
C12—O5	1.359 (3)	O5—H5O	0.825 (7)
C12—C13	1.386 (3)	O7—H7O	0.830 (7)
C12—C17	1.403 (3)	O1W—H1W	0.837 (7)
C13—C14	1.391 (4)	O1W—H2W	0.830 (7)
			
O1—C1—C2	124.0 (2)	C15—C14—C13	121.1 (2)
O1—C1—C6	118.8 (2)	C15—C14—H14	119.5
C2—C1—C6	117.2 (2)	C13—C14—H14	119.5
C1—C2—C3	121.9 (2)	O4—C11—O3	124.4 (2)
C1—C2—Br3	117.70 (18)	O4—C11—C8	124.0 (2)
C3—C2—Br3	120.31 (17)	O3—C11—C8	111.53 (19)
C4—C3—C2	120.5 (2)	C14—C15—C16	117.2 (2)
C4—C3—H3	119.7	C14—C15—C18	120.8 (2)
C2—C3—H3	119.7	C16—C15—C18	121.9 (2)
C3—C4—C5	118.1 (2)	O6—C20—O7	125.2 (2)
C3—C4—C7	121.3 (2)	O6—C20—C19	124.4 (2)
C5—C4—C7	120.5 (2)	O7—C20—C19	110.4 (2)
C6—C5—C4	120.5 (2)	N2—C19—C20	109.8 (2)
C6—C5—H5	119.8	N2—C19—C18	112.7 (2)
C4—C5—H5	119.8	C20—C19—C18	113.7 (2)
C4—C7—C8	115.08 (18)	N2—C19—H19	106.8
C4—C7—H7B	108.5	C20—C19—H19	106.8
C8—C7—H7B	108.5	C18—C19—H19	106.8
C4—C7—H7A	108.5	O8—C21—N2	121.2 (2)
C8—C7—H7A	108.5	O8—C21—C22	122.1 (2)
H7B—C7—H7A	107.5	N2—C21—C22	116.8 (2)
N1—C8—C11	109.89 (18)	C21—C22—H22C	109.5
N1—C8—C7	111.89 (19)	C21—C22—H22B	109.5
C11—C8—C7	111.97 (19)	H22C—C22—H22B	109.5
N1—C8—H8	107.6	C21—C22—H22A	109.5
C11—C8—H8	107.6	H22C—C22—H22A	109.5
C7—C8—H8	107.6	H22B—C22—H22A	109.5
O2—C9—N1	122.0 (2)	C15—C18—C19	116.3 (2)
O2—C9—C10	122.7 (2)	C15—C18—H18B	108.2
N1—C9—C10	115.3 (2)	C19—C18—H18B	108.2
C9—C10—H10C	109.5	C15—C18—H18A	108.2
C9—C10—H10A	109.5	C19—C18—H18A	108.2
H10C—C10—H10A	109.5	H18B—C18—H18A	107.4
C9—C10—H10B	109.5	C5—C6—C1	121.8 (2)
H10C—C10—H10B	109.5	C5—C6—Br1	119.52 (19)
H10A—C10—H10B	109.5	C1—C6—Br1	118.63 (18)
O5—C12—C13	124.2 (2)	C9—N1—C8	121.4 (2)
O5—C12—C17	119.0 (2)	C9—N1—H1N	122.9 (18)
C13—C12—C17	116.9 (2)	C8—N1—H1N	115.2 (18)
C12—C13—C14	121.7 (2)	C21—N2—C19	123.1 (2)
C12—C13—Br4	119.0 (2)	C21—N2—H2N	124.7 (19)
C14—C13—Br4	119.26 (18)	C19—N2—H2N	112.2 (19)
C16—C17—C12	121.7 (2)	C1—O1—H1O	113 (2)
C16—C17—Br2	120.17 (18)	C11—O3—H3O	106 (2)
C12—C17—Br2	118.09 (18)	C12—O5—H5O	115 (2)
C17—C16—C15	121.3 (2)	C20—O7—H7O	108 (2)
C17—C16—H16	119.3	H1W—O1W—H2W	108 (4)
C15—C16—H16	119.3		
			
O1—C1—C2—C3	−179.9 (2)	N1—C8—C11—O3	159.0 (2)
C6—C1—C2—C3	0.7 (3)	C7—C8—C11—O3	−76.0 (2)
O1—C1—C2—Br3	3.8 (3)	C13—C14—C15—C16	1.7 (3)
C6—C1—C2—Br3	−175.51 (16)	C13—C14—C15—C18	−175.2 (2)
C1—C2—C3—C4	−1.1 (3)	C17—C16—C15—C14	−1.7 (3)
Br3—C2—C3—C4	175.04 (17)	C17—C16—C15—C18	175.2 (2)
C2—C3—C4—C5	0.9 (3)	O6—C20—C19—N2	−0.1 (3)
C2—C3—C4—C7	−175.7 (2)	O7—C20—C19—N2	179.62 (19)
C3—C4—C5—C6	−0.3 (3)	O6—C20—C19—C18	127.2 (3)
C7—C4—C5—C6	176.3 (2)	O7—C20—C19—C18	−53.2 (3)
C3—C4—C7—C8	−98.5 (3)	C14—C15—C18—C19	−110.4 (3)
C5—C4—C7—C8	85.1 (3)	C16—C15—C18—C19	72.9 (3)
C4—C7—C8—N1	55.1 (3)	N2—C19—C18—C15	60.6 (3)
C4—C7—C8—C11	−68.8 (3)	C20—C19—C18—C15	−65.1 (3)
O5—C12—C13—C14	178.4 (2)	C4—C5—C6—C1	0.0 (3)
C17—C12—C13—C14	−2.2 (3)	C4—C5—C6—Br1	−176.88 (17)
O5—C12—C13—Br4	−2.3 (3)	O1—C1—C6—C5	−179.5 (2)
C17—C12—C13—Br4	177.10 (17)	C2—C1—C6—C5	−0.2 (3)
O5—C12—C17—C16	−178.3 (2)	O1—C1—C6—Br1	−2.6 (3)
C13—C12—C17—C16	2.3 (3)	C2—C1—C6—Br1	176.72 (16)
O5—C12—C17—Br2	0.6 (3)	O2—C9—N1—C8	2.6 (4)
C13—C12—C17—Br2	−178.86 (17)	C10—C9—N1—C8	−178.4 (2)
C12—C17—C16—C15	−0.3 (3)	C11—C8—N1—C9	−152.5 (2)
Br2—C17—C16—C15	−179.19 (17)	C7—C8—N1—C9	82.5 (3)
C12—C13—C14—C15	0.3 (4)	O8—C21—N2—C19	−5.7 (4)
Br4—C13—C14—C15	−179.05 (17)	C22—C21—N2—C19	173.3 (2)
N1—C8—C11—O4	−22.1 (3)	C20—C19—N2—C21	−135.0 (2)
C7—C8—C11—O4	102.9 (3)	C18—C19—N2—C21	97.2 (3)

### Hydrogen-bond geometry (Å, º)

**Table:**

*D*—H···*A*	*D*—H	H···*A*	*D*···*A*	*D*—H···*A*
N2—H2*N*···O1^i^	0.85 (1)	2.11 (1)	2.945 (3)	169 (3)
O7—H7*O*···O1*W*^ii^	0.83 (1)	1.78 (1)	2.607 (3)	171 (3)
O1*W*—H1*W*···O4^iii^	0.84 (1)	2.11 (2)	2.814 (3)	142 (3)
O1*W*—H2*W*···O2^iv^	0.83 (1)	2.01 (1)	2.836 (3)	172 (4)
N1—H1*N*···O5^v^	0.85 (1)	2.31 (1)	3.152 (3)	170 (2)
O3—H3*O*···O8^iv^	0.83 (1)	1.76 (1)	2.573 (2)	165 (3)
O5—H5*O*···Br4	0.83 (1)	2.73 (3)	3.1096 (16)	110 (2)
O5—H5*O*···O4^vi^	0.83 (1)	1.96 (1)	2.732 (2)	156 (3)
O1—H1*O*···O6^vii^	0.83 (1)	2.06 (2)	2.750 (2)	141 (3)
O1—H1*O*···Br3	0.83 (1)	2.61 (3)	3.0777 (17)	117 (2)

Symmetry codes: (i) −*x*+1, *y*+1/2, −*z*+1; (ii) *x*−1, *y*, *z*; (iii) *x*+1, *y*, *z*; (iv) *x*, *y*, *z*−1; (v) −*x*, *y*−1/2, −*z*+1; (vi) −*x*, *y*+1/2, −*z*+1; (vii) −*x*+1, *y*−1/2, −*z*+1.
